# Hepato-ocular crosstalk: Bile acids bridging pathogenesis and therapy

**DOI:** 10.1016/j.isci.2026.115118

**Published:** 2026-02-21

**Authors:** Peng Wang, Jingchen Xie, Feng Xiang, Suhui Xiong, Yamei Li, Bohou Xia, Limei Lin, Qiuxian Peng

**Affiliations:** 1 Key Laboratory for Quality Evaluation of Bulk Herbs of Hunan Province, School of Pharmacy, Hunan University of Chinese Medicine, Changsha 410208, China

**Keywords:** Health sciences

## Abstract

Bile acids, the major metabolites of cholesterol, function as pleiotropic signaling molecules beyond their classical role in lipid digestion. Increasing evidence indicates that dysregulated bile acid metabolism represents a shared molecular basis linking hepatic dysfunction with ocular pathology. Aberrations in bile acid synthesis, transport, and signaling lead to bile acid imbalance, which drives ocular injury through direct cytotoxicity, disruption of retinal and lens homeostasis mediated by FXR and TGR5 signaling, and immune activation along the gut-liver-eye axis. These mechanisms are implicated across a spectrum of conditions, ranging from inborn metabolic disorders to acquired cholestatic diseases. This review further highlights the translational potential of targeting bile acid homeostasis. We summarize emerging therapeutic strategies, including bile acid-based interventions, targeted drug delivery, and microbiome modulation, that aim to restore systemic bile acid balance. Collectively, we propose reconstruction of systemic bile acid homeostasis as a unifying therapeutic framework for hepato-ocular comorbidities.

## Graphical abstract

**Figure:**
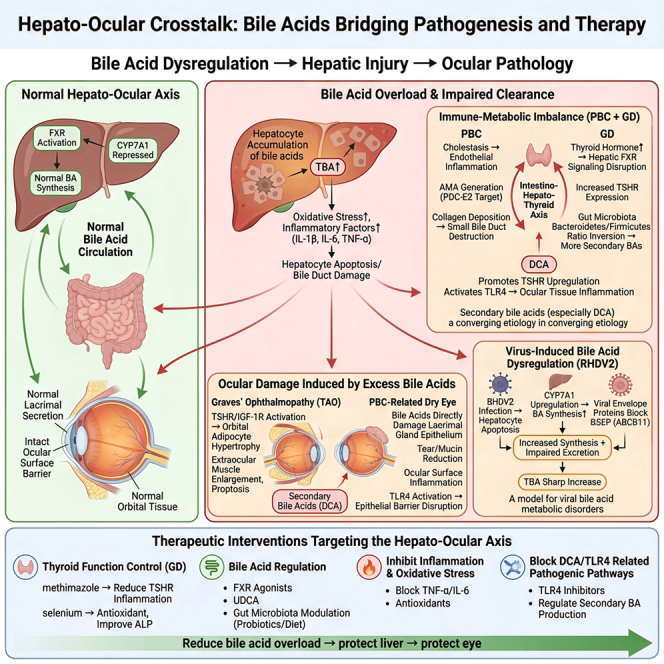

## Introduction

The liver, as the body’s central metabolic hub and primary detoxification organ, exerts profound influence on systemic homeostasis. Hepatic dysfunction not only drives metabolic derangements but also contributes to ocular pathology through diverse mechanisms.^1^ Clinical studies have demonstrated a strong association between nonalcoholic fatty liver disease (NAFLD) and ocular complications, including elevated intraocular pressure (IOP)^2^^,^^3^ and retinal microangiopathy, while patients with primary biliary cholangitis (PBC) frequently present with dry eye syndrome and keratoconjunctivitis.^4^ The underlying pathogenesis of these ocular manifestations involves impaired hepatic detoxification, immune-inflammatory activation,^5^ dysregulated lipid metabolism,^6^^,^^7^ and disruption of bile acid (BA) homeostasis,^8^ which collectively form the molecular framework of the “hepato-ocular axis.” Advances in mechanistic research have increasingly highlighted BAs as key mediators within this axis.

BA Physiology: Synthesis, Metabolism, and Enterohepatic Circulation. BAs are amphipathic molecules derived from cholesterol (CHOL), functioning both as detergents essential for lipid absorption and as signaling mediators.^9^ Hepatic synthesis generates the primary BAs, cholic acid (CA), and chenodeoxycholic acid (CDCA). In the intestine, microbial enzymes, particularly via 7α-dehydroxylation, convert these into secondary BAs—deoxycholic acid (DCA) from CA and lithocholic acid (LCA) from CDCA.^10^ This microbial conversion critically shapes the composition and bioactivity of the BA pool.^11^

BA homeostasis is maintained by the enterohepatic circulation, a highly efficient recycling system whereby ∼95% of intestinal BAs are reabsorbed in the ileum and returned to the liver via the portal vein. This process is tightly regulated by a negative feedback loop. In the ileum, BAs activate the nuclear receptor farnesoid X receptor (FXR), leading to release of fibroblast growth factor 19 (FGF19), which suppresses hepatic cholesterol 7α-hydroxylase (CYP7A1), the rate-limiting enzyme in BA synthesis. Concurrently, intrahepatic FXR activation induces the small heterodimer partner (SHP), further repressing CYP7A1 activity.^12^^,^^13^ Disruption of this circulation or its feedback control is a hallmark of BA dysregulation.

BA Transporters: The trafficking of BAs relies on dedicated transporters, and their dysfunction underlies many cholestatic disorders. The sodium-taurocholate cotransporting polypeptide mediates uptake of conjugated BAs into hepatocytes, while the bile salt export pump (BSEP) is the primary efflux transporter at the canalicular membrane. Under cholestatic conditions, the organic solute transporter α/β (OSTα/β) provides an alternative basolateral efflux pathway.^14^ Genetic mutations or pharmacological inhibition of these transporters, particularly BSEP, can lead to intrahepatic BA accumulation, cholestasis, and subsequent liver injury with systemic repercussions.

BA Receptors and Signaling: Beyond their digestive role, BAs function as signaling molecules through receptors such as FXR and the Takeda G Protein-Coupled Receptor 5 (TGR5). FXR, expressed abundantly in the liver, intestine, and kidney, regulates BA synthesis as well as lipid and glucose metabolism. TGR5, widely distributed in immune cells, adipose tissue, and the nervous system, modulates energy expenditure and exerts anti-inflammatory effects.^15^^,^^16^ Importantly, the presence of FXR and TGR5 in ocular tissues, including the retina and corneal epithelium, provides a direct molecular basis for BA-mediated hepatic-ocular crosstalk.

Taken together, BAs function as systemic regulators of lipid and glucose metabolism^17^^,^^18^ and as interorgan signaling mediators that integrate metabolic, immune, and epigenetic networks. Dysregulation of these pathways is a critical driver of liver disease pathogenesis^19^^,^^20^ and increasingly recognized in ocular pathology. Exogenous BA derivatives such as ursodeoxycholic acid (UDCA) not only reestablish intrahepatic BA balance but also confer ocular protection by attenuating apoptosis and enhancing antioxidant defenses. These insights support the development of targeted therapeutic strategies that exploit BA signaling to address hepato-ocular comorbidities.

## Molecular mechanisms by which BAS metabolic dysregulation mediates hepato-ocular pathological injury

### Pathological consequences of BAs metabolic blockade and accumulation

**Figure 1:**
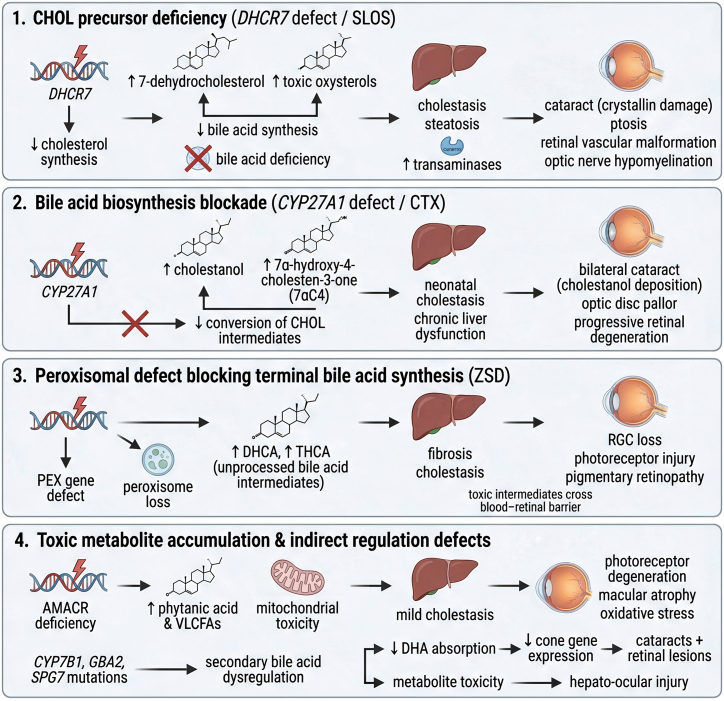
Hepato-ocular pathologies arising from impaired BA synthesis and toxic metabolite accumulation This figure illustrates the bidirectional pathological communication between the liver and ocular tissues under metabolic stress. Hepatic lipid accumulation, mitochondrial dysfunction, ER stress, and inflammatory activation promote systemic release of BA metabolites, cytokines, and oxidative mediators that impair ocular homeostasis. These circulating factors disrupt retinal vascular integrity, induce RGC apoptosis, trigger photoreceptor degeneration, and accelerate corneal epithelial inflammation. Conversely, ocular barrier breakdown and local inflammation feedback to exacerbate hepatic stress, forming a vicious cycle characteristic of hepato-ocular comorbidities.

Genetic defects affecting BA biosynthesis constitute a key mechanism underlying hepato-ocular injury. These disorders share a characteristic pathophysiological profile: impaired generation of functional BAs results in cholestasis and hepatic dysfunction, while the systemic accumulation of toxic intermediates directly compromises ocular structures (Figure 1, Panels 1–4).

#### Metabolic network dysregulation from biosynthetic defects and precursor deficiency

A critical vulnerability within this metabolic network lies in the synthesis of CHOL, the indispensable precursor of BAs. In Smith-Lemli-Opitz syndrome (SLOS), caused by mutations in dysfunctional 7-dehydrocholesterol reductase (DHCR7), impaired CHOL synthesis leads to reduced BA production. Clinically, this manifests as fat malabsorption, cholestasis, and hepatic abnormalities such as elevated transaminases and steatosis, which occur in approximately 25–29% of patients.^21^^,^^22^ Concomitantly, the metabolic blockade drives accumulation of 7-dehydrocholesterol and toxic oxysterols. These metabolites exert systemic toxicity, disrupting lens metabolism by inducing conformational changes in crystallin proteins, thereby promoting cataract formation. Additional ocular abnormalities—including ptosis, retinal vascular malformations, and optic nerve demyelination—highlight the multisystemic consequences of perturbed CHOL and BA homeostasis. This upstream precursor defect and its biochemical/ocular consequences correspond to Figure 1, Panel 1.

Beyond CHOL synthesis, defects in the enzymatic conversion of CHOL to mature BAs further illustrate this pathogenic paradigm. Cerebrotendinous xanthomatosis (CTX), resulting from sterol 27-hydroxylase (CYP27A1) deficiency, impairs the conversion of CHOL intermediates and leads to the accumulation of cholestanol. The ocular consequences are striking: bilateral cataracts develop in 76%–88% of affected individuals due to cholestanol deposition in the lens, which destabilizes lens proteins and promotes opacification.^23^^,^^24^ Hepatic manifestations include neonatal cholestatic jaundice and chronic liver dysfunction, reflecting the diminished levels of primary BAs such as CA and CDCA.^25^^,^^26^ In parallel, the deposition of toxic intermediates, particularly 7α-hydroxy-4-cholesten-3-one (7αC4), contributes to optic disc pallor and progressive retinal degeneration, thereby linking specific biochemical lesions to cumulative visual impairment.^25^ This mid-pathway enzymatic blockade corresponds to Figure 1, image 2.

#### Trans-organ toxicity from downstream metabolic and peroxisomal dysfunction

Pathological consequences of BA dysregulation also extend to organelles and enzymes that refine BA precursors. Peroxisomes are indispensable for the terminal steps of BA synthesis, mediating the conversion of dihydroxycholestanoic acid (DHCA) and trihydroxycholestanoic acid (THCA) into CDCA and CA.^27^ Of note, the retina contributes to systemic CHOL-BA metabolism by producing oxysterols in rod photoreceptors and retinal ganglion cells (RGCs), which are subsequently exported to the liver for further conversion.^27^ In Zellweger Syndrome Spectrum Disorder (ZSD), caused by pseudoxanthoma elasticum (PEX) mutations, loss of peroxisomal function halts this maturation process, resulting in hepatic fibrosis and cholestasis. The concomitant accumulation of BA precursors (e.g., DHCA and THCA) is believed to penetrate the blood-retinal barrier (BRB), directly injuring RGCs and photoreceptors, and giving rise to pigmentary retinal degeneration and optic nerve demyelination.^28^ These downstream peroxisomal dysfunctions correspond to Figure 1, image 3.

Enzyme deficiencies downstream of peroxisomal processing reveal additional layers of toxicity. Deficiency of α-methylacyl-CoA racemase (AMACR), an enzyme required for the metabolism of branched-chain fatty acids (e.g., phytanic acid), very long-chain fatty acids (VLCFAs), and BA intermediates, leads to systemic metabolite accumulation. Excess phytanic acid and VLCFAs induce mitochondrial toxicity and lipid peroxidation in retinal cells, resulting in pigment epithelial atrophy and macular degeneration.^29^ In parallel, impaired BA metabolism disrupts the absorption of essential fatty acids such as docosahexaenoic acid (DHA), thereby compromising cone photoreceptor function. Experimental models confirm that such metabolic perturbations downregulate cone-specific genes and accelerate photoreceptor degeneration via oxidative stress, illustrating how direct metabolite toxicity and nutritional deficiencies converge to drive retinal pathology.^30^ This mechanism of metabolite accumulation and reduced DHA absorption corresponds to Figure 1, image 4.

The complexity of genetic interaction networks further emphasizes the vulnerability of BA metabolism. Mutations in genes such as GBA2, oxysterol 7α-hydroxylase (CYP7B1), and SPG7 indirectly perturb BA homeostasis and give rise to overlapping clinical phenotypes, including hereditary spastic paraplegia accompanied by ocular manifestations such as cataracts and retinal lesions.^31^^,^^32^^,^^33^ These indirect regulatory defects are also incorporated within Figure 1, image 4 as secondary modulators contributing to hepato-ocular injury.

Collectively, these findings highlight that disruptions within the BA metabolic network, whether at the level of organelle function, enzymatic activity, or genetic regulation, represent a convergent pathogenic pathway linking hepatic and ocular disease.

### Pathological consequences of BAs overload and dysregulated clearance

In addition to inherited metabolic disorders, hepato-ocular injury can also result from acquired conditions characterized by BA overload, in which production surpasses clearance or excretion is impaired. Autoimmune and viral etiologies represent particularly illustrative models of this pathogenic paradigm.

#### Immune-metabolic imbalance-driven BAs overproduction and accumulation

**Figure 2:**
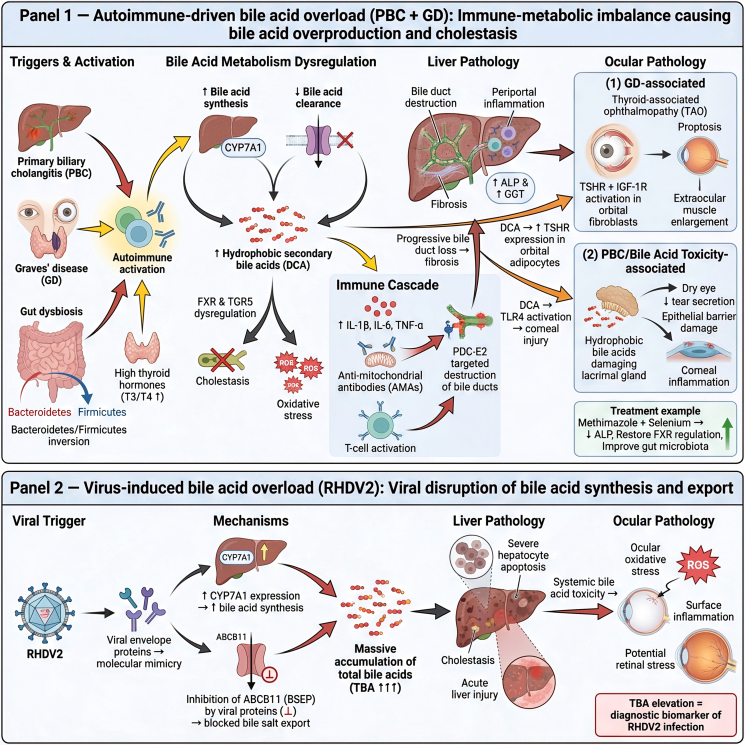
Hepato-ocular pathologies induced by BAs overproduction and impaired clearance BA synthesis, conjugation, transport, and enterohepatic circulation become markedly disrupted in chronic liver injury. Decreased BSEP/MRP2 activity and increased toxic hydrophobic BAs drive hepatocellular ER stress, oxidative damage, and apoptosis. Excess circulating BA species—particularly hydrophobic BA—penetrate ocular tissues, disrupting RPE tight junctions, promoting ROS accumulation, impairing photoreceptor-RPE phagocytic function, and contributing to corneal epithelial inflammation. Altered FXR/TGR5 signaling further amplifies metabolic imbalance and inflammation, linking BA toxicity to progressive hepato-ocular injury.

PBC provides a prototypical example of immune-mediated disruption of BA metabolism. The disease process is initiated by excessive intrahepatic BA accumulation, which drives inflammation through two interconnected mechanisms. Elevated BA concentrations directly stimulate hepatic sinusoidal endothelial cells to secrete proinflammatory cytokines such as IL-1β, IL-6, and TNF-α, thereby amplifying biliary injury and promoting fibrosis (Figure 2, image 1). Clinically, this response is reflected by increased serum alkaline phosphatase (ALP) and γ-glutamyl transferase levels.

In parallel, the inflammatory milieu contributes to a breakdown of immune tolerance, characterized by the production of anti-mitochondrial antibodies (AMAs) targeting the pyruvate dehydrogenase complex E2 in biliary epithelial cells. Together with dysregulated T cell responses and elevated expression of IFN-γ and TNF-α, this autoimmune cascade culminates in progressive bile duct destruction and fibrosis (Figure 2, image 1).^34^

The immune-metabolic imbalance in PBC demonstrates significant overlap with Graves’ disease (GD), suggesting the presence of an integrated “gut-liver-thyroid” axis. In patients with coexistent GD, elevated thyroid hormone levels further compromise immune tolerance to mitochondrial antigens, thereby accelerating AMA-mediated bile duct injury and disease progression (Figure 2, image 1).^35^ Hyperthyroidism also reshapes gut microbial composition, including inversion of the Bacteroidetes/Firmicutes ratio, which facilitates the conversion of primary to secondary BAs. These secondary metabolites, acting through FXR and TGR5 signaling, promote intrahepatic BA accumulation and oxidative stress, thereby establishing a self-reinforcing cycle that exacerbates cholestasis (Figure 2, image 1).^36^

This systemic pathophysiology manifests in distinct but mechanistically related ocular complications. In GD, thyroid-associated ophthalmopathy (TAO) is primarily mediated by thyroid-stimulating hormone receptor (TSHR)-driven activation of orbital fibroblasts and cross-reactivity with IGF-1R, resulting in proptosis and enlargement of extraocular muscles (Figure 2, image 1). In contrast, ocular surface disease associated with PBC, such as dry eye syndrome, arises from the direct cytotoxicity of hydrophobic BAs on the lacrimal glands. These BAs impair epithelial barrier integrity, reduce tear and mucin secretion, and trigger local inflammatory responses (Figure 2, image 1).^37^ Importantly, secondary BAs such as DCA may represent a convergent pathogenic factor. Experimental studies indicate that DCA enhances TSHR expression in orbital adipocytes and activates TLR4-mediated inflammatory damage in the cornea, thereby linking the ocular manifestations of both GD and PBC (Figure 2, image 1).^36^

From a therapeutic standpoint, this axis represents a promising target. Treatment of hyperthyroidism with methimazole, combined with selenium supplementation, not only improves thyroid function but also reduces serum ALP levels in patients with concomitant PBC and GD. These benefits are likely attributable to restoration of thyroid hormone-dependent regulation of FXR signaling and correction of gut dysbiosis (Figure 2, image 1).

#### Virus-associated BAs dysregulation and accumulation

Viral infections can directly interfere with hepatic BA homeostasis, leading to acute metabolic overload. Infection with rabbit hemorrhagic disease virus type 2 (RHDV2) illustrates this mechanism with particular clarity. As demonstrated by Bonvehí et al., RHDV2 induces hepatocellular apoptosis and upregulates expression of CYP7A1, the rate-limiting enzyme in BA synthesis, thereby increasing BA production. At the same time, viral envelope proteins act as molecular mimics that competitively inhibit ABCB11 (BSEP), the principal bile salt export pump. This combined strategy of “enhanced synthesis and impaired excretion” results in rapid and severe accumulation of total serum bile acids (TBAs) (Figure 2, image 2).^38^ The marked elevation of TBA not only establishes it as a specific biomarker for RHDV2 infection but also exemplifies how viral pathogens can precisely disrupt BA flux to drive both hepatic and systemic pathology.

### Pathological mechanisms of BAs transport disorders

The coordinated transport of BAs within the hepatobiliary system and their enterohepatic circulation is essential for maintaining systemic homeostasis. Disruption of these processes—whether due to genetic mutations or acquired conditions—results in BA mislocalization and accumulation. These changes exert direct cytotoxic effects and indirectly contribute to nutritional deficiencies, both of which have profound implications for ocular health.

#### Hereditary transporter defects and the spectrum of ocular manifestations

**Figure 3:**
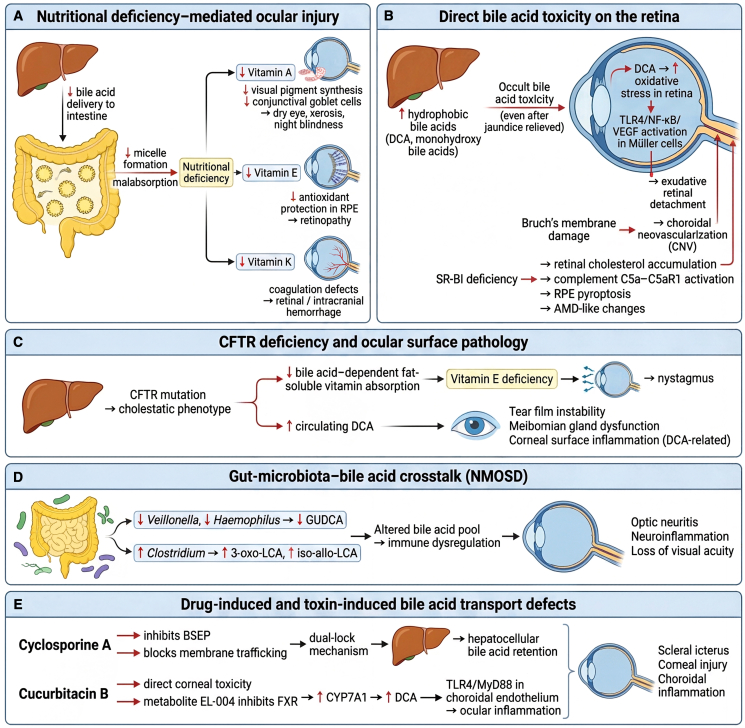
Hepato-ocular pathologies arising from BAs transport abnormalities This figure summarizes the protective outcomes of BA-based therapy across liver and ocular systems. (A) In hepatic tissues, TUDCA and UDCA reduce ER stress markers, restore BA transporters (BSEP and MRP2), inhibit apoptosis, and improve mitochondrial metabolic function. (B) In retinal and optic tissues, BAs stabilize RGC mitochondrial membrane potential, alleviate ROS production, and suppress photoreceptor degeneration. (C) In RPE and retinal vascular models, BAs enhance MerTK-mediated phagocytosis, protect barrier proteins such as ZO-1, and attenuate VEGF-driven angiogenic responses. (D) In corneal epithelial models, UDCA decreases inflammatory cytokines (CXCL8 and IL-5) and restores epithelial integrity. (E) Schematic summary demonstrating the integrated, multi-organ protection exerted by therapeutic BAs through ER stress suppression, antioxidant defense, anti-apoptotic signaling, and metabolic reconstitution.

Genetic disorders affecting BA transporters highlight the direct relationship between impaired hepatic excretion and ocular pathology. PEX, caused by mutations in ABCC6, exemplifies this link. Loss of ABCC6 function induces compensatory upregulation of other transporters such as ABCB11 (BSEP), reshaping the BA pool toward hydrophobic species like DCA. This alteration increases oxidative stress and has been clinically associated with choroidal neovascularization (CNV), likely through compromised integrity of Bruch’s membrane (Figure 3B).^39^

Progressive familial intrahepatic cholestasis (PFIC) represents a genetically heterogeneous group of transport defects with distinct hepatic and ocular phenotypes. Mutations in ATP8B1 (PFIC1), ABCB11 (PFIC2), ABCB4 (PFIC3), as well as syndromic forms such as JAG1 (Alagille syndrome, AGS) and CYP27A1 (THCA syndrome), demonstrate clear genotype-phenotype correlations in ocular involvement (summarized in Table 1). Clinical presentations range from macular cherry-red spots and albinotic fundus to severe ocular pruritus and posterior embryotoxon.

**Table 1:** Ocular phenotypic heterogeneity in patients with PFIC and related disorders

Subtypes	Pathogenic gene	Characteristic ocular symptoms	Pathogenesis	Reference
PFIC1	*ATP8B1*	bilateral macular “cherry-red spots” and Kayser-Fleischer rings	ATP8B1 mutations cause the aberrant expression of FIC1 protein in the macular region, leading to lipid deposition in the RGC layer and subsequent opacity of the extramacular retina	Fernández-Sánchez et al.^40^
PFIC2	*ABCB11*	albinotic fundus and tigroid/flecked retina	ABCB11 mutations result in BSEP deficiency, impairing BA transport at the bile canaliculi. This process disrupts fat absorption and induces fat-soluble vitamin (vitamins A, D, E, and K) deficiencies, ultimately compromising retinal function	Amirneni et al.^41^
PFIC3	*ABCB4*	ocular pruritus	ABCB4 mutations induce cholestasis, causing the systemic accumulation of BAs that stimulate cutaneous and ocular pruritus	Miethke et al.^42^
THCA syndrome	*CYP27A1*	retinal degeneration	CYP27A1 mutations lead to accumulation of the toxic BA precursor THCA	Fang et al.^43^
AGS	*AJG1*	posterior embryotoxon, choroidal sclerosis	JAG1 mutations impair Notch signaling pathway function, disrupting vascular and biliary duct development	Iboldet al.^39^

ATP8B1 encodes a P-type ATPase that transports aminophospholipids across cell membranes; mutations disrupt bile acid homeostasis. ABCB11 encodes the bile salt export pump (BSEP), critical for bile acid secretion at the canalicular membrane. ABCB4 encodes a phospholipid translocator (MDR3) involved in biliary phospholipid secretion. CYP27A1 encodes sterol 27-hydroxylase, a mitochondrial enzyme essential for bile acid biosynthesis. JAG1 encodes Jagged1, a ligand in the Notch signaling pathway involved in development of bile ducts and ocular structures.

These manifestations arise either from direct BA toxicity (Figure 3B) or, more commonly, from malabsorption of fat-soluble vitamins secondary to reduced intestinal BA availability (Figure 3A). Specifically, vitamin A deficiency (Figure 3A) leads to impaired synthesis of visual pigments and a reduction in conjunctival goblet cell count, resulting in night blindness and dry eye disease. Vitamin K (Figure 3A) deficiency can cause coagulation dysfunction, increasing the risk of intracranial and retinal hemorrhages, while vitamin E (Figure 3A) deficiency reduces retinal antioxidant capacity and contributes to retinopathy.^41^^,^^42^

An important concept emerging from PFIC is that of “occult BA toxicity.” Even following surgical interventions that alleviate jaundice, persistently elevated serum concentrations of monohydroxy BAs (e.g., 3β-hydroxy-5-cholenoic acid) may sustain ocular injury (Figure 3B). Two mechanisms are implicated.

(1) Vitamin E deficiency leading to lipid peroxidation within the retinal pigment epithelium (RPE), thereby reducing the retina’s antioxidant capacity and contributing to retinopathy (Figures 3A and 3B);
(2) Direct stimulation of the TLR4/NF-κB/VEGF signaling cascade in Müller cells, which promotes exudative retinal detachment (Figure 3B).^40^^,^^43^

Cystic fibrosis, caused by cystic fibrosis transmembrane conductance regulator (CFTR) mutations, also exemplifies the ocular consequences of impaired BA transport. The resulting cholestatic phenotype disrupts the absorption of fat-soluble vitamins, with vitamin E deficiency linked to nystagmus. Additionally, BA imbalance alters tear film composition, predisposing to meibomian gland dysfunction and corneal surface disease, where clinical severity correlates with circulating DCA concentrations (Figure 3C).

Collectively, these conditions underscore that defects in BA transport extend beyond hepatic cholestasis, systemically impairing nutritional support and destabilizing ocular structures essential for retinal integrity and ocular surface homeostasis.

#### Acquired transport disorders, drug-induced dysregulation, and gut-microbiota crosstalk

In addition to genetic defects, BA transport can be impaired by acquired conditions, pharmacological agents, and alterations in the gut microbiota, each of which exerts distinct yet interconnected effects on ocular health.

Chronic hepatic congestion exemplifies how systemic disease can disrupt BA transport. Reduced reabsorption of BAs through FXR-mediated downregulation of OSTα/β leads to deficiencies in fat-soluble vitamins, particularly vitamin A and vitamin E, which are indispensable for photoreceptor function and protection of the RPE from oxidative stress (Figures 3A and 3E).^44^

Transporter dysfunction may also arise from drug exposure. Drug-induced cholestasis frequently occurs when lipophilic compounds such as cyclosporine A or their metabolites inhibit BSEP directly or interfere with its membrane trafficking. This “dual-lock mechanism” results in hepatocellular BA retention, cholangiocyte apoptosis, and clinically manifests as scleral icterus (Figure 3E).^45^ Similarly, environmental toxins such as cucurbitacin B exhibit dual toxicity: they exert direct corneal injury while their metabolite EL-004 suppresses hepatic FXR activity. The consequent upregulation of CYP7A1 promotes elevated serum DCA, activating the TLR4/MyD88 pathway in choroidal endothelial cells and amplifying ocular inflammation (Figure 3E).

The gut-liver axis further modulates BA transport and toxicity. In neuromyelitis optica spectrum disorder (NMOSD), dysbiosis characterized by reduced Veillonella and Haemophilus abundance diminishes glycoursodeoxycholic acid (GUDCA) production, while enrichment of Clostridium species increases production of 3-oxo-LCA and iso-allo-LCA, contributing to immune dysregulation associated with optic neuritis (Figure 3D).^46^

In metabolic syndrome, impaired reverse CHOL transport—exemplified by SR-BI deficiency—leads to retinal CHOL accumulation, initiating RPE pyroptosis via activation of the complement C5a-C5aR1 axis and promoting pathological changes resembling age-related macular degeneration (AMD) (Figure 3B).^47^ Nutritional interventions, including astaxanthin-rich extracts, may counteract such processes by modulating BA metabolism at multiple regulatory nodes (e.g., LXRα, FXR, and ABCG5/G8) and reshaping gut microbiota composition.^48^

The clinical implications of these mechanisms are considerable. Patients with PFIC require long-term ophthalmological surveillance to detect progressive retinal changes; CFTR modulators have been reported to improve ocular surface health in cystic fibrosis (Figure 3C); and combined supplementation with vitamin E and UDCA offers potential benefit in restoring retinal function and mitigating oxidative stress-related injury.^49^

### Shared serum BA features in clinical patients with hepato-ocular disorders

Clinical investigations support the mechanistic link between BA dysregulation and hepato-ocular injury, demonstrating that distinct liver diseases are associated with characteristic alterations in serum BA profiles. These “BA signatures” not only represent potential biomarkers but also shed light on the pathophysiological basis of ocular complications, as summarized in Table 2.

**Table 2:** Summary of BAs dysregulation patterns and their associated ocular manifestations in hepato-ocular disorders

Pattern of BA dysregulation	Primary associated liver condition(s)	Characteristic ocular manifestation(s)	Proposed key pathogenic mechanisms linking liver to eye	Category/evidence level	Reference
Deficiency of functional BAs and precursor toxicity	SLOS (DHCR7 mutation)	cataracts, ptosis, retinal vascular malformations, optic nerve demyelination	1. toxic precursor accumulation (7-DHC, oxysterols) causing lens protein misfolding 2. systemic deficiency of BA leads to fat-soluble vitamin malabsorption	inborn error of metabolism (clinical and genetic)	Nowaczyk et al.^21^
	CTX (CYP27A1 deficiency)	bilateral cataracts, optic disc pallor, progressive retinal degeneration	1. cholestanol deposition in lens 2. toxic intermediate (7αC4) accumulation affecting retina	inborn error of metabolism (clinical and genetic)	Salen et al.^25^
	ZSD/AMACR deficiency	pigmentary retinal degeneration, macular degeneration, photoreceptor loss	1. direct retinal toxicity from accumulated VLCFAs/phytanic acid 2. nutritional deficiency (e.g., DHA) impairing photoreceptor function	inborn error of metabolism/peroxisomal disorder	Chen et al.^27^
BA overload and altered composition	PBC	dry eye syndrome, keratoconjunctivitis	1. direct cytotoxicity of hydrophobic BAs on lacrimal glands and ocular surface 2. immune-inflammatory axis shared with autoimmune epithelitis	autoimmune cholangiopathy (clinical and serologic)	Gulamhusein et al.^34^
	GD (with PBC)	TAO	1. shared immune dysregulation (TSHR/IGF-1R) 2. secondary BAs (e.g., DCA) enhancing TSHR expression and corneal inflammation via TLR4	immune-metabolic axis	Acharya et al.^35^
	Viral infection (e.g., RHDV2 model)	(model for systemic BA overflow)	“Synthesis↑ + Excretion↓” leading to acute systemic BA overload, demonstrating potential for vascular/ocular toxicity	acquired, viral model (preclinical)	Bonvehí et al.^38^
Transport defect with vitamin deficiency	PFIC (multiple subtypes)	macular cherry-red spots, albinotic fundus, ocular pruritus, posterior embryotoxon	1. direct BA toxicity on retinal cells 2. malabsorption of fat-soluble vitamins (A, E, and K) leading to night blindness, retinopathy, and hemorrhagic risk	genetic transport disorder (clinical and genetic; Table 1)	Ibold et al.^39^
	cystic fibrosis (CFTR)	nystagmus, meibomian gland dysfunction, corneal disease	1. vitamin E deficiency due to cholestasis 2. altered tear film from systemic BA imbalance	systemic disease with cholestasis	Schneider-Futschik et al.^49^
Specific serum BA “signature”	NAFLD/DILI	POAG, acute angle-closure glaucoma	1. elevated total and primary BAs impairing trabecular meshwork function 2. TLR4/NF-κB activation in anterior segment 3. gut microbiota (megamonas) dysbiosis	clinical metabolomic association	Seo et al.^50^
	NAFLD/ALD	hepatogenic retinopathy (microvascular abnormalities)	1. elevated CDCA potentially activating retinal FXR/VEGF 2. elevated TUDCA as a biomarker/mediator of BRB disruption with context-dependent effects	clinical-biochemical correlation	Xie et al.^51^
	chronic HCV, cirrhosis	retinopathy (hemorrhages, vascular tortuosity)	1. elevated TCA activating retinal NLRP3 inflammasome 2. TCA inhibiting eNOS, reducing retinal perfusion	clinical-metabolomic and mechanistic	Smith et al.^52^
	chronic HBV, HCC	exudative AMD	1. deficiency of GUDCA due to HBV-induced CYP7A1 suppression 2. loss of GUDCA’s endogenous anti-angiogenic (anti-CNV) protection	epidemiological and preclinical interventional	Warden et al.^53^

#### Elevated TBAs: A metabolic nexus linking fatty liver and drug-induced injury to glaucoma

Clinical observations consistently link systemic BA overload to glaucoma. Epidemiological data indicate a robust association between fatty liver disease (e.g., NAFLD) and elevated IOP.^50^ This is further substantiated by findings that patients with both NAFLD and primary open-angle glaucoma (POAG) exhibit significantly elevated TBAs compared to controls.^54^ Supporting the role of a gut-liver-eye axis, a marked reduction in Megamonas species—a bacterium implicated in BA metabolism—has been reported in both POAG and NAFLD patients, correlating with disease severity.^55^ Additional clinical evidence comes from cases of drug-induced liver injury (DILI), where acute hepatotoxicity (e.g., from Garcinia cambogia or levofloxacin) coincides with pronounced TBA elevations and episodes of acute angle-closure glaucoma.^56^ Notably, serum BA patterns in DILI and POAG patients show striking similarities, characterized by a disproportionate increase in primary BAs.^51^

To explain how systemic BA overload may elevate IOP, preclinical studies point to direct effects on ocular tissues. Mechanistic investigations suggest that circulating BAs, accumulated due to impaired hepatic excretion, can impair phagocytic function in trabecular meshwork cells and activate the proinflammatory TLR4/NF-κB signaling pathway in corneal cells. Together, these effects are hypothesized to increase aqueous humor outflow resistance and possibly stimulate its production, thereby contributing to IOP elevation and glaucomatous progression.^55^

#### Aberrant increases in CDCA and TUDCA: Metabolic biomarkers of hepatogenic retinopathy

Clinical studies have identified specific BA elevations in patients with liver disease-associated retinopathy. Epidemiological evidence indicates a higher prevalence and severity of retinopathy in patients with NAFLD and alcohol-related liver disease (ALD).^57^ A characteristic biochemical signature in these patients is the elevation of serum CDCA and tauroursodeoxycholic acid (TUDCA).^58^ Furthermore, within ALD cohorts, TUDCA concentrations are often higher in patients with retinopathy compared to those without ocular involvement, suggesting its potential as a biomarker for BRB disruption.^59^

The pathogenic implications of these elevations are explored through mechanistic hypotheses. For CDCA, experimental models suggest its potential to activate retinal FXR, leading to upregulation of VEGF, which could contribute to retinal microvascular dysfunction and pathological angiogenesis.^60^ Regarding TUDCA, while its rise may initially represent an endogenous cytoprotective response, preclinical data indicate a potential paradoxical effect under pathological conditions. At supraphysiological levels, TUDCA has been shown in experimental settings to destabilize mitochondrial membrane potential in retinal Müller cells, implying a context-dependent role that might transition from compensatory to potentially detrimental in advanced disease.^61^

#### TCA-specific elevation: A characteristic alteration in viral hepatitis and cirrhosis-associated retinopathy

A distinct clinical signature emerges in retinopathy associated with chronic viral hepatitis and cirrhosis. Patients with chronic hepatitis C virus (HCV) infection and cirrhosis exhibit a markedly increased incidence of retinal hemorrhages and microvascular abnormalities. Metabolomic profiling consistently identifies taurocholic acid (TCA) as significantly elevated in these patient cohorts compared to healthy individuals. Advanced imaging analyses have further revealed a positive correlation between retinal vascular tortuosity and circulating TCA levels, strengthening its biomarker potential.^62^

Preclinical mechanistic studies propose that TCA is not merely a biomarker but an active pathogenic mediator. Two principal pathways have been investigated: First, TCA can activate the NLRP3 inflammasome, enhancing IL-1β secretion and thereby amplifying retinal inflammation. Second, TCA inhibits eNOS, leading to impaired nitric oxide production, diminished retinal perfusion, and increased susceptibility to ischemic injury.^52^ These mechanisms position elevated TCA as a potential therapeutic target in this setting.

#### Reduced GUDCA levels: A metabolic link between HBV-HCC and macular degeneration

In contrast to the elevations described above, a deficiency of a specific BA links hepatic and ocular pathology. Epidemiological studies demonstrate that individuals with chronic hepatitis B virus (HBV) infection or HBV-associated hepatocellular carcinoma (HCC) have a significantly increased risk of developing exudative AMD and disease progression.^63^ Corroborating this link, clinical studies consistently report significantly lower circulating levels of GUDCA in patients with HBV-related HCC. This deficiency is mechanistically attributed to the suppression of the rate-limiting enzyme CYP7A1 by the HBV X protein.^64^

The protective role of GUDCA, suggested by its deficiency in patients, is strongly supported by interventional preclinical models. Supplementation with exogenous GUDCA markedly attenuates CNV in murine models of AMD, highlighting its role as an endogenous cytoprotective agent.^53^ These findings suggest that GUDCA deficiency constitutes a critical metabolic mechanism underlying the shared susceptibility to AMD in HBV-infected and HCC patients, positioning GUDCA replacement as a potential therapeutic strategy.

## Targeting BAS metabolism for the treatment of hepato-ocular comorbidities

### Exogenous BAs replacement therapy: Restoring metabolic homeostasis across organ systems

The therapeutic rationale for modulating BA metabolism in hepato-ocular comorbidities is strongly supported by clinical and experimental evidence demonstrating the efficacy of exogenous BA administration. Supplementation with selected BAs not only corrects endogenous deficiencies but also counterbalances the cytotoxic effects of hydrophobic BA species, thereby contributing to the restoration of systemic metabolic homeostasis. Through these mechanisms, exogenous BA therapy has the potential to attenuate pathological processes affecting both hepatic and ocular systems. Nonetheless, the biological actions of certain BAs, such as UDCA, can be complex and occasionally paradoxical, underscoring the need for detailed mechanistic insights to fully define their therapeutic value and limitations. The principal clinical applications and mechanistic bases of BA therapeutics are summarized in Table 3.

**Table 3:** Investigational and approved roles of supplemental BAs in disorders with hepato-ocular manifestations

Supplemented BA	Associated hepatic disorder(s)	Associated/potential ocular disorder(s)	Proposed or established mechanism of action	Notes	Reference
TUDCA	studied in PBC, cirrhosis (not a first-line therapy for cholestasis)	dry eye syndrome, retinopathy (based on mechanistic studies)	acts as a chemical chaperone to mitigate ER stress; modulates oxidative stress and inflammatory responses. Increases hydrophilicity of the BA pool	clinical utility is primarily supported by older and/or mechanistic studies; not standard care for indicated liver disorders	Atlıhan et al.^65^
UDCA	PBC (FDA-approved), gallstones (FDA-approved) off-label use explored in: PFIC, CF-associated liver disease, select DILI, NAFLD	retinal damage, visual impairment (exploratory clinical and preclinical evidence)	displaces cytotoxic BAs; enhances biliary excretion; stabilizes hepatocyte membranes. Shifts BA pool toward hydrophilicity. Demonstrates ability to cross the BRB in models	first-line therapy for PBC. Benefits in other hepatic and ocular disorders are investigational/off-label	Andreazzoliet al.^66^

#### TUDCA: A metabolic regulator with hepato-ocular protective potential

While not a first-line therapy for cholestatic disorders, TUDCA has been extensively studied as a potent cytoprotective agent in experimental models. Its protective effects are mediated through multiple mechanisms: in preclinical studies, TUDCA has been shown to improve hepatic biochemical indices, shift the BA pool toward more hydrophilic species, and stabilize hepatocyte membranes against the cytotoxicity of hydrophobic BAs. Data from older clinical studies suggest that in cirrhotic patients, TUDCA administration was associated with reductions in cholestasis markers and improvements in some histopathological features. More importantly, robust preclinical evidence underscores its potential for extrahepatic protection. Experimental studies in models of chronic liver disease have demonstrated that TUDCA can mitigate pathological processes relevant to ocular complications, such as reducing systemic inflammation and oxidative stress.^65^ This promising dual-organ protective profile in mechanistic studies highlights TUDCA’s potential as a cytoprotective agent worthy of further clinical investigation in the context of hepato-ocular comorbidities.

#### UDCA: Multidimensional modulation of hepato-ocular health

UDCA is the first-line, FDA-approved therapy for PBC and is also used for the dissolution of CHOL gallstones. Its well-characterized mechanisms—including displacement of cytotoxic BAs, enhancement of biliary excretion, and stabilization of hepatocyte membranes—underpin its clinical utility in these conditions. Beyond its approved indications, UDCA has been investigated for its potential benefits in a range of other hepatic and extrahepatic disorders, supported by its diverse pharmacological actions.

In the context of metabolic and cholestatic diseases, UDCA’s mechanisms may address specific pathophysiological features. For instance, in CTX, the underlying defect leads to abnormal BA synthesis and cholestanol accumulation. While UDCA is not a specific therapy for CTX, its general cholestasis-alleviating properties have been explored. Some studies suggest that UDCA may improve biochemical parameters, though its effect on the neurological and ocular manifestations (like retinal changes) requires further validation.^67^ In NAFLD, UDCA has been studied for its potential to ameliorate cholestasis and oxidative stress, leading to improvements in liver enzymes.^68^ It is important to note that these uses in CTX and NAFLD are considered off-label.

Similarly, UDCA has been utilized off-label in several other disorders characterized by cholestasis or BA-mediated injury. In PFIC, it can improve biochemical parameters and reduce symptoms like hepatosplenomegaly.^69^ In chronic viral hepatitis and cystic fibrosis-associated liver disease, UDCA treatment has been associated with modulation of oxidative stress and immune responses, though its impact on disease progression remains an area of study. Of particular interest for hepato-ocular crosstalk, UDCA treatment in cystic fibrosis has been linked to improved visual contrast sensitivity in some reports, potentially related to systemic hepatoprotection and improved nutritional status.^66^

UDCA is also an established option in the management of certain forms of DILI.^70^ Furthermore, its potential role in preserving hepatic function and possibly modulating carcinogenesis in cirrhosis has been explored.

A key feature supporting its investigation in ocular conditions is its ability to cross the BRB. Preclinical studies demonstrate that UDCA exerts direct cytoprotective effects on retinal cells, including photoreceptors, through anti-apoptotic, antioxidant, and anti-inflammatory mechanisms.^71^

Despite its broad investigational use, the clinical application of UDCA outside of PBC and gallstones requires careful consideration. Furthermore, a paradoxical aspect of UDCA biology exists: elevated endogenous levels may correlate with disease states like retinopathy, whereas pharmacologic administration aims to achieve cytoprotective effects. This highlights the complexity of BA signaling.

Finally, while UDCA’s substitutive and cytoprotective actions are valuable, they primarily mitigate injury rather than correct the underlying pathogenic drive in many disorders. This has spurred the development of next-generation agents (e.g., FXR agonists) designed to directly modulate BA synthesis and excretion, representing a complementary therapeutic strategy.

#### Dose dependence and long-term safety of BA therapy

Although BA-based therapy has accumulated decades of clinical experience in hepatic diseases, the optimization of dosing strategies and assessment of long-term safety remain active challenges, particularly in pediatric, pregnant, and multisystemic populations. These issues involve intricate pharmacokinetic variability, receptor sensitivity, and gut microbiota interactions that collectively shape therapeutic outcomes.

Clinical evidence for dose-dependent effects highlights strong disease specificity. In patients with bile acid synthesis disorders (BASDs), oral CA at 10–15 mg/kg/day significantly reduces urinary atypical BA excretion, normalizes serum aminotransferases, and improves growth indices. This range activates hepatic FXR signaling to repress CYP7A1 expression and limit hepatotoxic intermediates, while concomitantly stimulating TGR5 to promote hepatocyte regeneration. In contrast, in PBC, the optimal response to UDCA occurs at 13–15 mg/kg/day; suboptimal dosing (<10 mg/kg/day) leads to incomplete biochemical response in approximately 40% of patients, evidenced by persistently elevated ALP and increased risk of disease progression.^72^ Insufficient UDCA exposure fails to competitively inhibit hydrophobic BA cytotoxicity and inadequately activates the Nrf2 antioxidant pathway.^73^

Cystic fibrosis-associated liver disease (CFLD) illustrates another aspect of dose dependency. In a long-term study of 20 CFLD patients, high-dose UDCA (20 mg/kg/day) over eight years did not result in LCA accumulation (serum <0.05 μmol/L), countering earlier toxicity concerns.^74^ The absence of LCA buildup may reflect reduced bacterial 7α-dehydroxylation capacity in CFLD microbiota, suggesting that gut ecological context modulates the threshold of safe dosing. Similarly, patients with Δ^4^-3-oxosteroid 5β-reductase deficiency exhibit individualized dose requirements for CDCA (5.5–10 mg/kg/day), guided by urinary 3-oxo-Δ^4^ BA levels; excessive suppression can impair BA synthesis and lipid absorption.^75^

Long-term safety evaluation has primarily focused on hepatic accumulation risk and systemic metabolic impact. In pediatric BASD cohorts, CA therapy followed for up to 18 years revealed no irreversible hepatic injury or growth retardation, with stable bilirubin and coagulation profiles. Correspondingly, in Abcb4^−/−^ mice, long-term administration of the FXR agonist INT-767 (15 months) reduced collagen deposition and inflammatory cytokines (TNF-α and IL-6) while improving fibrosis, though the protective effect was lost in FXR-deficient animals—emphasizing receptor integrity as a prerequisite for safety.^76^

UDCA’s long-term tolerability is well established in PBC, whereas the newer FXR agonist obeticholic acid (OCA) shows dose-dependent adverse reactions. In the POISE trial, 5–10 mg/day OCA reduced ALP by 39%–47%, but pruritus incidence increased from 60% (1.5–3 mg/day) to 67% (5–10 mg/day).^77^ This pruritus is primarily mediated through the activation of MRGPRX4, a key receptor in humans that binds to BAs and initiates the itch sensation.^78^ More severe outcomes, including hyperbilirubinemia and hepatic decompensation, have been observed in cirrhotic patients (Child-Pugh B/C), likely due to FXR overactivation-mediated downregulation of the BSEP.^79^

Dosing strategies in special populations require careful pharmacokinetic adjustment. In children with PFIC, a phase II trial of the ileal BA transporter inhibitor odevixibat demonstrated a dose-response relationship between 10 and 200 μg/kg/day and serum BA reduction (r = −0.72, *p* < 0.001); the 100 μg/kg/day dose achieved a 2.8-point reduction in pruritus without growth impairment. This dose achieved ∼70% reduction in enterohepatic BA reuptake while minimizing systemic exposure.^79^

Comprehensive monitoring frameworks are essential for long-term management.

(1) Biochemical—monthly TBA and ALP monitoring, quarterly urinary atypical BA profiling;
(2) Imaging—annual liver elastography to track fibrosis;
(3) Molecular—serum FGF19 assessment, with sustained reduction (<100 pg/mL) indicating feedback suppression failure.^80^

In cystic fibrosis, fecal LCA > 0.1 μmol/g indicates dysbiosis and may warrant probiotic co-therapy.^74^

Collectively, these findings emphasize that optimizing BA therapy demands a precision-medicine framework integrating disease context, receptor biology, and microbiome status to balance efficacy with long-term safety.

### Molecular mechanisms of BAs therapeutics: Integrated cytoprotection across hepatic and ocular tissues

**Figure 4:**
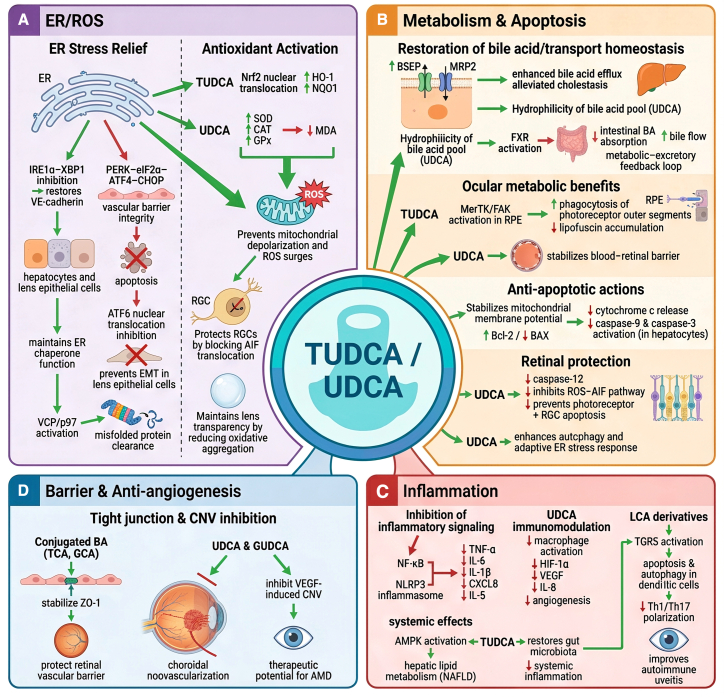
The therapeutic mechanisms of BAs treatment in hepato-ocular disorders A centralized schematic summarizes four major modules of TUDCA/UDCA-mediated cytoprotection across liver and ocular tissues. (A) ER stress suppression and antioxidant defense: TUDCA modulates all three UPR branches (IRE1α-XBP1, PERK-ATF4-CHOP, and ATF6), activates VCP/p97-dependent protein clearance, and enhances Nrf2 signaling. UDCA increases antioxidant enzyme activity (SOD, CAT, and GPx) and reduces MDA, preventing ROS surges, AIF translocation, and oxidative protein aggregation. (B) Metabolic restoration and anti-apoptotic integration: BAs upregulate BSEP/MRP2, enhance bile flow via FXR signaling, and increase BA hydrophilicity. Ocular benefits include MerTK/FAK-driven phagocytosis and BRB stabilization. Anti-apoptotic actions include improved mitochondrial membrane potential, increased Bcl-2, decreased BAX, and suppression of caspase pathways. (C) Anti-inflammatory and immunomodulatory actions: TUDCA inhibits NF-κB and NLRP3, reducing TNF-α, IL-6, IL-1β, and ocular inflammatory mediators. UDCA suppresses macrophage activation and angiogenic factors (HIF-1α, VEGF, and IL-8). LCA derivatives activate TGR5 to inhibit Th1/Th17 polarization. (D) Retinal vascular barrier protection and anti-angiogenesis: Conjugated BAs (TCA and GCA) stabilize ZO-1 and preserve retinal vascular integrity. UDCA and GUDCA inhibit VEGF-induced CNV, highlighting therapeutic potential for AMD and angiogenesis-driven retinopathies.

The therapeutic benefits of BAs such as TUDCA and UDCA derive from their pleiotropic capacity to modulate fundamental cellular stress pathways central to the pathogenesis of both hepatic and ocular diseases. Rather than targeting a single molecular process, these compounds restore cellular and tissue homeostasis through a coordinated network of cytoprotective mechanisms. Key actions include the suppression of endoplasmic reticulum (ER) stress and oxidative injury, the reconstruction of disrupted metabolic and transport networks, the inhibition of apoptosis, and the modulation of inflammatory responses (summarized in Figure 4).

#### Core cellular stress mitigation: ER stress suppression and antioxidant defense

One of the principal mechanisms by which BAs exert cytoprotective effects is the alleviation of ER stress, a critical driver of metabolic dysfunction. As illustrated in Figure 4A, TUDCA functions as a chemical chaperone, modulating all three branches of the unfolded protein response (UPR). It inhibits the IRE1α-XBP1 signaling axis, stabilizing endothelial junction proteins such as VE-cadherin and thereby restoring vascular barrier integrity in models of diabetic retinopathy (DR).^81^ In parallel, it attenuates activation of the PERK-eIF2α-ATF4-CHOP pathway, reducing CHOP-mediated apoptosis in hepatocytes exposed to rifampicin (RFP) toxicity as well as in lens epithelial cells, ultimately mitigating ER stress-driven cell death and fibrotic remodeling. TUDCA also modulates ATF6 activity by preventing its nuclear translocation, thereby supporting ER chaperone function and preserving proteostasis. This action helps maintain lens epithelial cell integrity and prevents epithelial-mesenchymal transition.^82^ These effects are reinforced by enhanced clearance of misfolded proteins through activation of the VCP/p97 complex and by the preservation of intercellular junction stability via modulation of O-GlcNAcylation.^83^

Complementing ER stress suppression, BAs also activate endogenous antioxidant defense systems (Figure 4A, right image). TUDCA promotes nuclear translocation of Nrf2, leading to increased expression of antioxidant enzymes such as HO-1 and NQO1.^82^ UDCA enhances the activity of key antioxidant enzymes, including SOD, CAT, and GPx, while reducing levels of lipid peroxidation markers such as MDA.^84^ In hepatic tissues, these effects protect against toxin-induced mitochondrial depolarization and ROS surges.^82^ In ocular tissues, activation of these antioxidant pathways promotes the survival of RGCs by preventing apoptosis-inducing factor (AIF) translocation, and it maintains lens transparency by counteracting oxidative protein aggregation.^85^

#### Metabolic network reconstitution and anti-apoptotic integration

In addition to alleviating cellular stress, therapeutic BAs play a pivotal role in re-establishing metabolic and transport homeostasis (Figure 4B). They remodel hepatic BA handling by upregulating efflux transporters such as the BSEP and multidrug resistance-associated protein 2 (MRP2), thereby facilitating BA clearance and alleviating cholestasis. UDCA further enhances the hydrophilicity of the BA pool, diminishing overall cytotoxicity. Moreover, activation of nuclear receptors—particularly the FXR—by TUDCA and CA establishes a “metabolic-excretory feedback loop,” suppressing intestinal BA reabsorption and promoting bile flow.^86^

In ocular tissues, these systemic metabolic effects are complemented by organ-specific actions. TUDCA enhances the phagocytic activity of RPE cells via the MerTK/FAK signaling pathway, a process essential for the daily clearance of photoreceptor outer segments and the prevention of toxic lipofuscin accumulation. UDCA contributes additional benefits by stabilizing the BRB, an effect mediated in part through its anti-inflammatory properties.^87^

These metabolic improvements are closely coupled with robust anti-apoptotic mechanisms. TUDCA stabilizes mitochondrial membrane potential by modulating the Bcl-2/BAX ratio, thereby preventing cytochrome *c* release and the subsequent activation of caspase-9 and caspase-3 in hepatocytes.^86^ In the retina, TUDCA inhibits ER stress-associated caspase-12 activation and blocks the ROS-AIF translocation pathway, conferring protection to RGCs and photoreceptors in experimental models of retinal detachment and degeneration.^88^ UDCA acts synergistically by promoting autophagic clearance of damaged organelles and proteins, while upregulating adaptive ER stress response genes, thereby enhancing overall cellular resilience.^89^

#### Multidimensional anti-inflammatory and immunomodulatory actions

The protective effects of BAs are reinforced by their broad-spectrum anti-inflammatory and immunomodulatory activities (Figure 4C). TUDCA exerts marked inhibitory effects on central proinflammatory signaling pathways, notably NF-κB and the NLRP3 inflammasome. Through this dual mechanism, TUDCA reduces the production of key cytokines such as TNF-α, IL-6, and IL-1β in hepatic systems, while concurrently suppressing CXCL8 and IL-5 expression in corneal epithelial cells, thereby attenuating inflammatory responses in both liver and ocular tissues.^90^ UDCA further contributes to immunoregulation by dampening macrophage activation and tumor angiogenesis through downregulation of HIF-1α, VEGF, and IL-8.

Distinct BA species also exert selective immunomodulatory functions. For example, LCA derivatives modulate dendritic cell biology by inducing apoptosis and autophagy in bone marrow-derived dendritic cells (BMDCs) through TGR5 activation, thereby inhibiting Th1/Th17 polarization and ameliorating autoimmune uveitis (Figure 4D).^91^

These molecular mechanisms collectively converge on the maintenance of tissue homeostasis. UDCA facilitates hepatic lipid metabolism in NAFLD through AMPK activation,^84^ while TUDCA promotes gut microbiota restoration, indirectly mitigating systemic inflammatory signaling.^91^ Conjugated BAs, such as TCA and glycocholic acid (GCA), safeguard the retinal vascular barrier by stabilizing tight junction proteins, including ZO-1, and compounds like GUDCA and UDCA directly suppress VEGF-induced CNV, highlighting their therapeutic promise for angiogenesis-driven ocular disorders, such as AMD (Figure 4D).^92^

Taken together, the therapeutic efficacy of BAs does not derive from a single pathway but from their coordinated engagement of multiple, interlinked defense systems. By simultaneously alleviating ER and oxidative stress, restoring metabolic homeostasis, inhibiting cell death pathways, and suppressing inflammation, BAs provide an integrated strategy to counteract the complex, shared pathophysiological mechanisms underlying hepato-ocular comorbidities. A schematic overview of these mechanisms is presented in Figure 4, while the detailed molecular pathways are summarized in Table 4.

**Table 4:** Key preclinical evidence linking BAs cytoprotection in hepato-ocular pathobiology

Supplemented BA	Research Model	Key Mechanisms & Targets	Main Findings & Relevance to Hepato-Ocular Axis	Reference
TUDCA	*in vivo*: mouse cholestasis model (ANIT) *in vitro*: RFP-induced HepG2 cells	FXR, Nrf2 activation; UPR/ER stress inhibition (↓PERK-CHOP, ↓IRE1α); ↑BSEP/MRP2	alleviates cholestatic liver injury and protects retinal cells. Demonstrates shared cytoprotective pathways (FXR, Nrf2, and UPR) active in both hepatocytes and retinal cells, supporting a common mechanistic basis for therapy	Zhang et al.^82^
	*in vivo*: P23H rat (retinal degeneration) *in vitro*: ARPE-19 cells	UPR inhibition (↓GRP78, ↓CHOP); modulation of sphingolipid metabolism	mitigates systemic ER stress and retinal apoptosis. Shows that a BA therapeutic can ameliorate retinal degeneration in a model of systemic disease, linking protein misfolding stress across organs	Afşar et al.^83^
	*in vivo*: diabetic (STZ) rats *in vitro*: high glucose-induced HRMECs	NF-κB pathway inhibition; antioxidant effects	ameliorates DR. Illustrates how BA therapy targeting systemic inflammation and oxidative stress (common in liver disease) can directly improve diabetic retinal microvascular damage	Dai et al.^81^
	*in vitro*: ARPE-19 & hRPE cells *in vivo*: rd10 mouse, P23H rat	activation of MerTK/FAK phagocytic pathway	rescues RPE phagocytic function. Connects BA signaling to a critical retinal homeostatic process (photoreceptor outer segment clearance), disrupted in both retinal and metabolic liver diseases	Daruich et al.^87^
UDCA	*in vivo*: STZ-induced diabetic mice *in vitro*: retinal pericytes	UPR suppression (↓GRP78, ↓pPERK); Anti-inflammatory (↓MCP-1, TNF-α)	preserves retinal vascular integrity in diabetes. Demonstrates direct retinal vascular protection via mechanisms (UPR, inflammation) also central to its hepatoprotective action in cholestasis	Wang et al.^84^
	*in vivo*: bile duct ligated Abcb11^−/−^ mice *ex vivo*: mouse models	FXR-dependent regulation of detoxification genes (↑Cyp3a11, Mrp4)	promotes hepatic detoxification and bile flow. While primarily a hepatic model, it elucidates the FXR-mediated “metabolic reprogramming” mechanism that underlies systemic toxin clearance, indirectly benefiting retinal health	Song et al.^86^
	*in vivo*: light-induced retinal degeneration mice	anti-apoptotic pathway (↓caspase-3)	directly protects photoreceptors from degeneration. Provides proof-of-concept that UDCA crosses the BRB to exert cytoprotection, analogous to its membrane-stabilizing effect in hepatocytes	Zhang et al.^88^
LCA derivative	*in vivo*: experimental autoimmune uveitis mouse *in vitro*: BMDCs	TGR5 activation → induces DC apoptosis/autophagy; inhibits NF-κB/MAPK	modulates systemic immunity to suppress ocular autoimmunity. Highlights a BA receptor (TGR5)-mediated immune regulatory axis that originates in immune cells but can attenuate organ-specific inflammatory eye disease	Hu et al.^91^

#### Integrated model of BAs signaling in hepato-ocular crosstalk

BA signaling represents a convergent regulatory framework that coordinates metabolic, inflammatory, and stress-response networks across hepatic and ocular systems. The integrated actions of FXR, TGR5, and S1PR2—together with the intestinal FXR-FGF19 endocrine loop—form a multidimensional signaling axis that maintains systemic and local homeostasis through inter-organ communication.^93^ Dysregulation of this axis generates synchronized metabolic and inflammatory disturbances, establishing the “shared vulnerability” of hepatocytes and retinal cells.

At the core of this network, FXR functions as a nuclear transcriptional hub linking BA metabolism with cytoprotective gene expression. In hepatocytes, FXR activation induces SHP and represses CYP7A1 to suppress BA synthesis, while enhancing efflux via BSEP and MRP2 to prevent cholestatic stress.^94^ In ocular tissues, FXR activation in retinal pigment epithelial and vascular endothelial cells inhibits NF-κB signaling and induces antioxidant enzymes (SOD and GPx), thereby maintaining redox and barrier integrity.^95^ Beyond local effects, intestinal FXR activation stimulates FGF19 secretion, which binds FGFR4/β-Klotho complexes in liver and retina to initiate ERK, JNK, and AKT cascades—suppressing CYP7A1 and improving glucose and lipid metabolism while enhancing mitochondrial function.^96^

TGR5, a membrane-bound G protein-coupled receptor, complements FXR by translating extracellular BA fluctuations into intracellular cAMP-PKA signals that mediate anti-inflammatory and metabolic effects.^97^ In hepatic Kupffer and cholangiocyte populations, TGR5 activation attenuates TNF-α, IL-1β, and IL-6 release and promotes bile flow, thereby mitigating cholestatic and metabolic injury. In retinal neurons and Müller glia, TGR5 signaling suppresses NLRP3 inflammasome activation and ROS generation, preserving neuronal survival and BRB integrity.^96^ Through cAMP-PKA-CREB activation, TGR5 also enhances Nrf2-mediated antioxidant responses and mitochondrial biogenesis, creating a shared cytoprotective program across both tissues.^98^

S1PR2 provides an additional layer of integration by coupling BA signaling to sphingolipid and kinase cascades. In hepatocytes and cholangiocytes, S1PR2 activation engages ERK1/2 and PI3K/AKT pathways to promote cell survival and metabolism under physiological conditions, whereas excessive stimulation drives NF-κB-dependent inflammation and fibrosis. Similarly, in the retina, S1PR2 contributes to vascular leakage and endothelial activation; pharmacologic inhibition of this receptor restores barrier integrity in diabetic and ischemic retinopathies by suppressing ERK/NF-κB signaling.^96^ Hence, S1PR2 functions as a context-dependent rheostat, balancing adaptive and maladaptive responses to BA stress.

At the mechanistic intersection of these receptors lies the control of oxidative, ER, and mitochondrial stress. FXR upregulates Nrf2-driven antioxidant enzymes (SOD, CAT, and GPx) and inhibits NADPH oxidase-mediated ROS production, while TGR5 reinforces these effects through cAMP-PKA-Nrf2 signaling.^98^ Both receptors mitigate ER stress by enhancing chaperone expression (GRP78 and GRP94) and inhibiting PERK-eIF2α-CHOP-driven apoptosis.^99^ Together, they preserve mitochondrial integrity by promoting PGC-1α-mediated biogenesis, regulating fission/fusion dynamics, and facilitating mitophagy.^100^ In contrast, aberrant S1PR2 signaling can amplify oxidative and ER stress through JNK/NF-κB activation, underscoring its dual role in stress adaptation.^95^ These receptor-mediated mechanisms are mirrored in retinal neurons and pigment epithelial cells, where FXR and TGR5 activation alleviate oxidative and ER stress and sustain mitochondrial activity under pathologic conditions such as DR and light-induced injury.^96^

Integratively, the FXR-TGR5-S1PR2-FGF19 axis constitutes a unified hepato-ocular signaling model in which BA receptors orchestrate redox balance, metabolic remodeling, and inflammatory control across both organs. Perturbations at any node—BA overload, receptor desensitization, or microbiome dysbiosis—disrupt this equilibrium, yielding parallel hepatic and retinal injury phenotypes including steatosis, cholestasis, and neurovascular degeneration.^101^ Conversely, pharmacologic activation or selective modulation of these receptors restores BA homeostasis and cross-organ protection, providing a mechanistic rationale for multi-receptor-targeted BA therapeutics aimed at preserving systemic metabolic integrity.

Collectively, this integrated model reconciles previously fragmented findings into a unified biological logic: BA receptors operate as a coordinated inter-organ defense network whose failure defines the molecular basis of hepato-ocular comorbidities.

## Conclusion and future direction

The intricate interplay between hepatic and ocular health is critically governed by BA metabolism, which serves as a central regulatory axis integrating metabolic, immunological, and microbial signals. Dysregulation at any level of this network—whether in synthesis, transport, or receptor-mediated signaling—can destabilize systemic homeostasis, simultaneously predisposing both the liver and the eye to pathological injury. Current evidence supports a unifying paradigm: hepato-ocular comorbidities are not incidental associations but arise from shared mechanisms, mediated by the systemic circulation of BAs and the overlapping expression of BA receptors across hepatic and ocular tissues.

### Converging mechanisms of hepato-ocular injury

The pathogenic mechanisms underlying hepato-ocular comorbidities are multifactorial yet tightly interconnected.^23^ Genetic defects in BA synthesis (e.g., SLOS and CTX) or transport (e.g., PFIC and ABCC6 deficiency) result in a dual insult characterized by insufficient functional BA pools and the accumulation of toxic intermediates.^31^ These perturbations directly impair retinal cells and lens proteins, leading to ocular dysfunction.^29^ In acquired conditions, such as PBC and viral hepatitis, pathological BA overload initiates a cascade of oxidative stress and inflammatory signaling, notably through NF-κB and NLRP3 activation, which compromise hepatobiliary integrity while simultaneously disrupting ocular barriers, including the lacrimal gland and the BRB.^102^

Beyond intrinsic hepatic or ocular pathology, the gut-liver-eye axis emerges as a critical amplifier. Dysbiosis alters BA composition and receptor signaling, thereby reshaping systemic immune responses and predisposing to inflammatory conditions such as optic neuritis.^46^ Collectively, whether triggered by genetic mutations, immune dysregulation, or microbial imbalance, the final converging pathway frequently involves BA-driven metabolic stress and inflammation, underscoring BAs as pivotal mediators in the shared pathophysiology of hepato-ocular disorders.^46^

### Knowledge gaps and current limitations

Despite compelling preclinical and associative clinical data, several critical limitations must be acknowledged to accurately frame the current state of the field. Most notably, there is a pronounced scarcity of direct human clinical data. The majority of evidence linking specific BA signatures to ocular outcomes remains correlative, derived from cohort studies, and causal relationships are often inferred from animal models.^103^ Furthermore, rigorous, prospective clinical trials evaluating BA-targeted therapies (e.g., UDCA and FXR agonists) for the specific prevention or treatment of ocular complications in liver disease patients are largely lacking.^104^ The field also grapples with significant heterogeneity; individual variations in gut microbiota composition,^105^ genetic background,^106^ and disease stage can dramatically influence both BA profiles and therapeutic responses, complicating the development of universal biomarkers or treatment protocols.^107^ Addressing these gaps requires future research to prioritize longitudinal human studies that integrate deep phenotyping with multi-omics profiling to establish causality and identify predictive biomarkers.

### Therapeutic implications and a shift to multidimensional intervention

The identification of BAs as active pathogenic mediators has provided a framework for targeted therapeutic strategies. Exogenous supplementation with agents such as UDCA and TUDCA has demonstrated that restoration of BA homeostasis can attenuate ER stress, apoptosis, and inflammatory signaling, thereby exerting beneficial effects on both hepatic and ocular systems.^63^ However, the translation of these therapies requires careful consideration of potential risks. The dose-dependent pruritus associated with FXR agonists like obeticholic acid, mediated through receptors such as MRGPRX4, is a well-known adverse effect that can limit tolerability. More serious risks, including hepatic decompensation in advanced cirrhosis, highlight the necessity for patient stratification and monitoring. Furthermore, the paradoxical, context-dependent effects of certain BAs (e.g., potential neurotoxicity of supraphysiological TUDCA) underscore that their therapeutic window and long-term safety profile in extrahepatic tissues require further clarification.^108^

#### Precision drug delivery via novel nanocarriers

Nanotechnology-based delivery platforms represent a transformative advance for enhancing the efficacy and specificity of BA-derived therapies. Exploiting the intestinal apical sodium-dependent BA transporter (ASBT) can facilitate selective hepatic uptake of therapeutic agents, improving bioavailability and reducing systemic toxicity.^109^ More sophisticated approaches employ ligand-functionalized nanocarriers, such as galactose (Gal)-modified UDCA-polypropylene glycol (UDCA-PPG), which enable coordinated targeting of hepatic and ocular tissues. These systems have been shown to activate cytoprotective pathways, including Nrf2/HO-1, while concurrently suppressing NF-κB-driven inflammatory cascades.^110^

Further structural refinements, such as polyethylene glycol or polyethyleneimine (PEI) modification, enhance solubility, stability, and therapeutic activity. In bile duct ligation models, such modifications have been associated with regulation of apoptosis-related markers (Bax/Bcl-2 and caspase-3) and autophagy regulators (LC3 and p62), underscoring their potential in mitigating cholestatic injury.^111^ Moreover, the development of dual-targeting systems—exemplified by Gal-modified nanostructured lipid carriers (Gal-NLC)—offers the capacity to simultaneously accumulate at hepatic and retinal sites of injury. Such platforms may provide a novel therapeutic paradigm for the integrated management of hepato-ocular comorbidities, combining metabolic restoration with localized cytoprotection.^112^

#### Restoring homeostasis through microbiome remodeling

The gut microbiota plays a pivotal role in regulating the BA pool, and therapeutic strategies aimed at microbiome remodeling hold significant promise. Fecal microbiota transplantation (FMT) has shown efficacy in ameliorating nonalcoholic steatohepatitis (NASH) by reconstructing a balanced intestinal microbial community.^112^ Its potential is currently under investigation in clinical trials targeting NAFLD, NASH, and cirrhosis (NCT03803540, NCT02469272, and NCT02721264). In parallel, BA sequestrants such as colesevelam improve systemic metabolic parameters by reducing the BA pool size, resulting in decreased body weight, lower LDL CHOL, and attenuated hepatic steatosis.^113^ A combined approach that integrates FMT to restore beneficial microbial populations with sequestrants to reshape the biochemical milieu may synergistically correct BA dysmetabolism, providing a novel therapeutic avenue for the management of liver-eye comorbidities.

In summary, the concept of a “hepato-ocular axis” is firmly supported by BA biology. Advancing this field requires not only precise characterization of BA signatures associated with specific ocular pathologies but also a clear-eyed assessment of current evidence limitations and therapeutic risks. The development of future therapeutic strategies must integrate precision targeting with microbiome modulation while adhering to rigorous safety pharmacovigilance. Such multidimensional and cautious approaches offer the potential to move beyond symptomatic management toward the restoration of the metabolic equilibrium that fundamentally links the liver and the eye.

## Acknowledgments

We thank BioRender for providing drawing support and AJE for English language editing. This work was supported by the 10.13039/501100001809National Natural Science Foundation of China (82474151); the Discipline Construction Project of Hunan University of Chinese Medicine (Z2023JBGS05); the Key Project of Education Bureau of Hunan Province (23A0293); and the Hunan Provincial Key Research and Development Program (2023SK4001).

## Author contributions

P.W., investigation and writing – original draft; J.X., writing – review and editing; F.X., methodology; S.X., visualization and software; Y.L., visualization and software; B.X., conceptualization; L.L., project administration and funding acquisition; Q.P., conceptualization and supervision.

## Declaration of interests

The authors declare no competing financial interests.
